# Familial cardiac laminopathy with predominant atrial involvement: a case series of a family with *LMNA* mutation

**DOI:** 10.1093/ehjcr/ytaf129

**Published:** 2025-03-15

**Authors:** Andreas Müssigbrodt, Romain Vergier, Maria Herrera Bethencourt, Astrid Monfort, Jocelyn Inamo, Veronique Fressart, Pascale Richard, Patrice Bouvagnet

**Affiliations:** Department of Cardiology, CHU Martinique (University Hospital of Martinique), BP 632, 97200 Fort de France, France; Department of Cardiology, CHU Martinique (University Hospital of Martinique), BP 632, 97200 Fort de France, France; Department of Cardiology, CHU Martinique (University Hospital of Martinique), BP 632, 97200 Fort de France, France; Department of Cardiology, CHU Martinique (University Hospital of Martinique), BP 632, 97200 Fort de France, France; Department of Cardiology, CHU Martinique (University Hospital of Martinique), BP 632, 97200 Fort de France, France; Unité Fonctionnelle de Cardiogénétique et Myogénétique Moléculaire et Cellulaire, DMU Biogem, Service de Biochimie Métabolique, AP-HP-Sorbonne Université, Pitié-Salpêtrière - Charles Foix, 47-83, boulevard de l’Hôpital, 75651 Paris cedex 13, France; Unité Fonctionnelle de Cardiogénétique et Myogénétique Moléculaire et Cellulaire, DMU Biogem, Service de Biochimie Métabolique, AP-HP-Sorbonne Université, Pitié-Salpêtrière - Charles Foix, 47-83, boulevard de l’Hôpital, 75651 Paris cedex 13, France; Department of Cardiology, CHU Martinique (University Hospital of Martinique), BP 632, 97200 Fort de France, France

**Keywords:** Laminopathy, LMNA, Mutation, Atrial fibrillation, Case report

## Abstract

**Background:**

We present a case series detailing a family with familial cardiac laminopathy, including the female index patient, her father, her brother, and her daughter, all diagnosed with atrial arrhythmias, i.e. atrial fibrillation (AF), atrial flutter, and atrial premature contractions.

**Case summary:**

The index patient is known for frequent premature atrial contractions since the age of 35 years and for persistent AF since the age of 46 years. Rhythm control of symptomatic AF was achieved after two ablations. The brother of the index patient is known for asymptomatic persistent AF since the age of 26 years. He has opted for rate control and oral anticoagulation. Their father had persistent AF for many years before his death. The daughter of the index patient has asymptomatic frequent premature atrial contractions. She is under medical surveillance without specific medical treatment. All four patients have a normal ventricular systolic function. Genetic testing revealed a heterozygous missense variant in the *LMNA* gene causing a familial form of cardiac laminopathy, with clinical predisposition to atrial arrhythmias.

**Discussion:**

The familial occurrence of atrial arrhythmias without significant ventricular involvement in several family members with heterozygous *LMNA* missense mutation underscores the potential genetic component in cardiac arrhythmias, especially if arrhythmias occur in younger individuals without common risk factors.

**Learning points**

- This case report highlights atrial arrhythmias (premature atrial contraction and atrial fibrillation) as the main manifestation of a *LMNA* mutation.
- Possible indications for anticoagulation and rhythm control are discussed in the context of current evidence and guidelines.

## Introduction

Atrial fibrillation (AF) can be associated with a genetic predisposition, particularly in younger individuals without common risk factors.^1,2^ Familial laminopathies are well known for cardiac involvement, with heart failure as well as atrial and ventricular arrhythmias as the main manifestations.^1,2^ We describe the clinical presentation and management of atrial arrhythmias in a family with *LMNA* mutation, highlighting the importance of genetic counselling and surveillance in hereditary cardiac conditions.

## Case presentation

### Index patient

**Figure 1:**
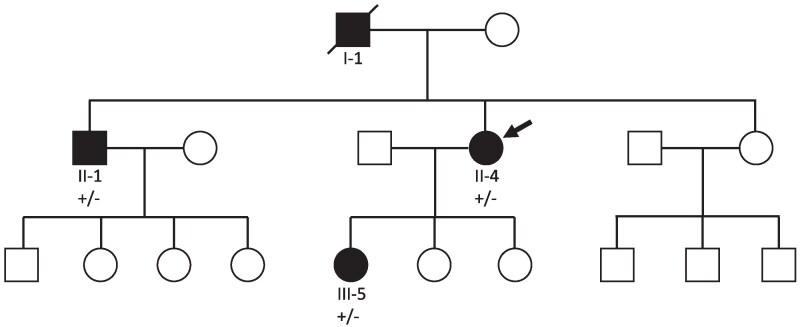
A pedigree tree is presented. Squares and circles represent males and females, respectively; open symbols denote healthy; filled symbols denote affected; arrow denotes proband; diagonal line denotes deceased.

The female index patient (*Figure 1, II-4*), previously only known for hypothyroidism with levothyroxine substitution, was firstly diagnosed with frequent premature atrial contractions (PAC) at the age of 35 years. She is a mother of three children. She had a regular follow-up (FU) by a general physician and an occasional FU by a cardiologist. An echocardiogram—done at the age of 42 years—was normal. Her height and weight are normal (168 cm, 55 kg, BMI 19.5). Her medical treatment consisted of flecainide, levothyroxine, and oral contraception.

A first stroke with right-sided paresis occurred at the age of 45 years. Electrocardiogram (ECG) at hospital admission showed sinus tachycardia at 112/min. Transthoracic echocardiography demonstrated normal cardiac dimensions and a normal left ventricular ejection fraction (LVEF) of 58%. Neurological symptoms resolved within a few days. An ambulatory Holter ECG described her cardiac rhythm as sinus rhythm (SR) with PAC.

A second stroke with right-sided paresis and aphasia occurred at the age of 46 years. At that time, she was treated with aspirin, flecainide, perindopril, and levothyroxine. At hospital arrival, ECG confirmed AF. Cerebral magnetic resonance imaging (MRI) demonstrated an occluded left proximal middle cerebral artery (MCA). Interventional thrombectomy was performed. Motor symptoms resolved after thrombectomy. Transthoracic echocardiography was normal. At discharge, the medical treatment consisted of apixaban, atorvastatin, flecainide, and perindopril.

**Figure 2:**
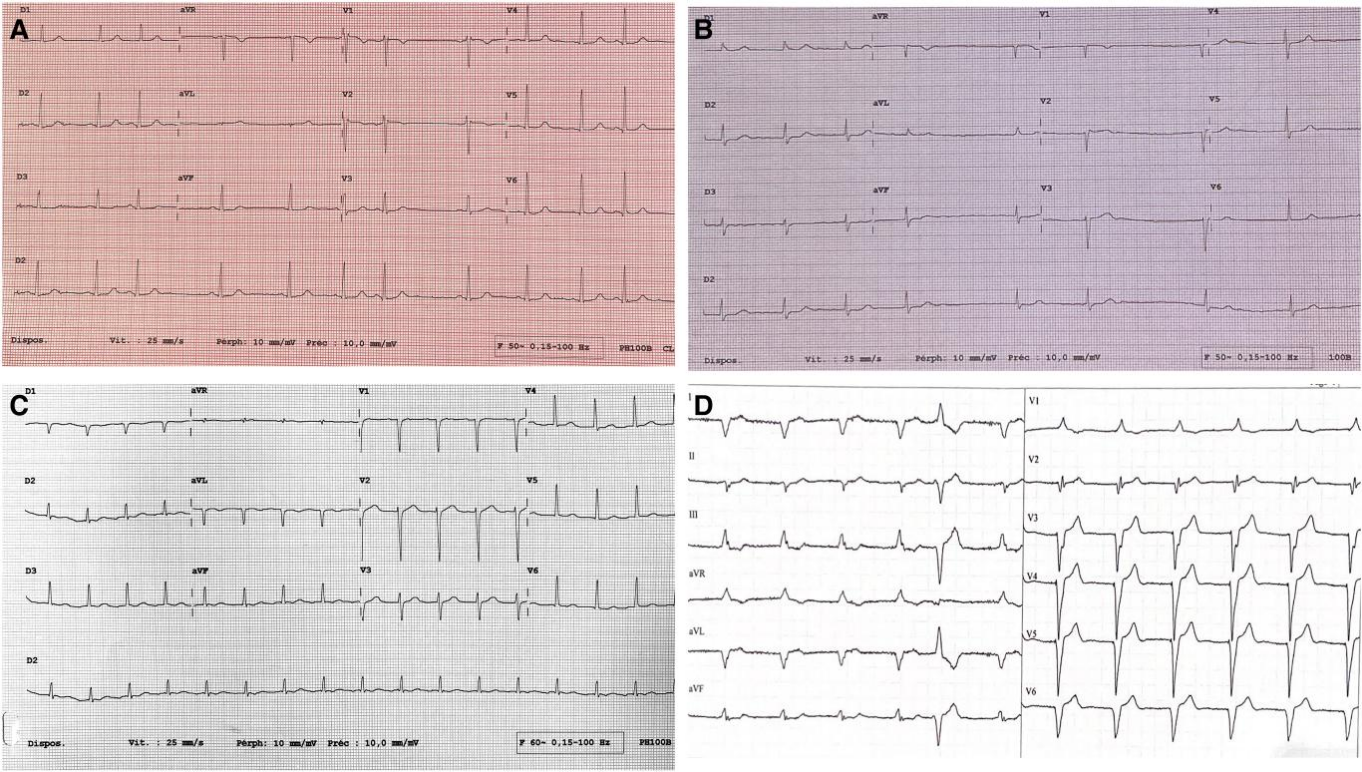
Electrocardiogram of the four family members with different stages of atrial disease progression. (*A*) Daughter of the index patient: sinus rhythm 70/min, PR 160 ms, QRS 80 ms, QTc 430 ms, and premature atrial contractions. (*B*) Brother of the index patient: atrial fibrillation, heart rate 54/min, QRS 109 ms, and QTc 420 ms. (*C*) Index patient (before second ablation) left atrial flutter with 2:1 conduction, heart rate 100/min, and QRS 100 ms. (*D*) Father of the index patient: atrial fibrillation, biventricular pacing at 70/min, QRS 146 ms, and one premature ventricular contraction.

**Figure 3:**
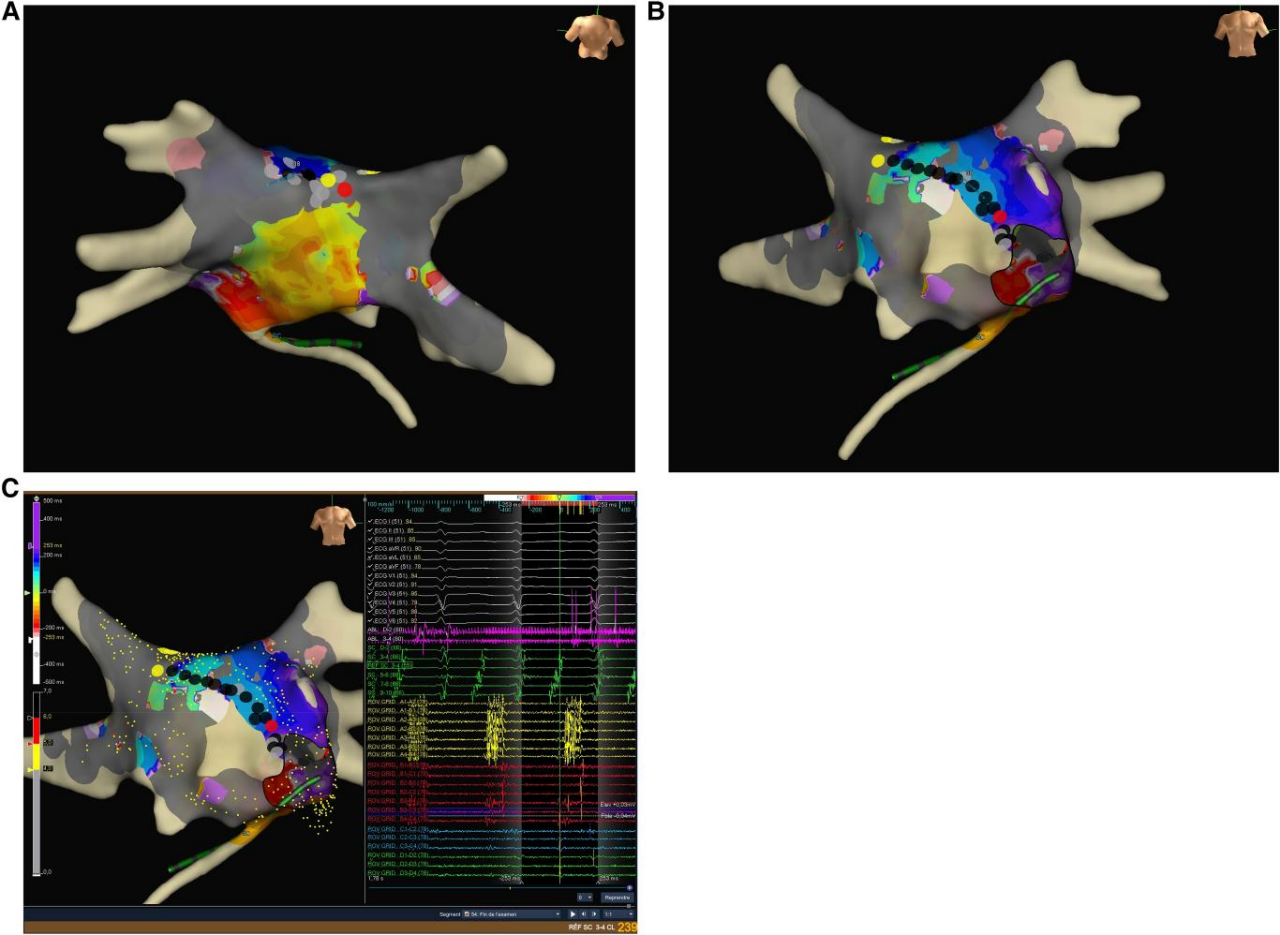
Electroanatomic mapping of the second ablation procedure of the index patient demonstrates scarring and left atrial flutter as manifestations of atrial cardiomyopathy: the first colour-coded activation map (*A*: posterior view) demonstrates a left atrial flutter around the left pulmonary veins with a zone of slow conduction at the left atrial roof between the right superior pulmonary vein and left superior pulmonary vein. Grey-coloured pulmonary vein represents lack of electrical activity inside the pulmonary veins after pulmonary vein isolation at the first ablation procedure. The grey areas between the right superior pulmonary vein and left superior pulmonary vein, along with the inferior septum, demonstrate atrial scarring, indicating atrial myopathy. Ablation between the left and right superior pulmonary vein (black, yellow, and red dots) converted the first atrial flutter into another flutter around the mitral valve annulus, with a septal slow conduction zone (*B*: anterior view; *C*: anterior view) with electrocardiogram and electrograms from a decapolar catheter in the coronary sinus and from a multipolar catheter (ROV) at the septum. Ablation between the anterior mitral valve and the right superior pulmonary vein (black, yellow, and red dots) terminated the second atrial flutter, with subsequent evidence of bidirectional block across both lines, isolated pulmonary veins, and no further inducibility of arrhythmias with burst pacing and programmed stimulation during isoproterenol infusion.

In the outpatient clinic, she described mild palpitations. The patient agreed to an electrical cardioversion in order to better understand the role of AF in her symptoms. As she felt improved symptoms in SR after the electrical cardioversion, she agreed to undergo ablation. A pulmonary vein isolation was performed at the age of 46 years. Voltage map of the left atrium showed an altered voltage with fragmented signals as a sign of diseased atrial myocardium. A second ablation was performed at the age of 47 years due to symptomatic left atrial flutter (*Figures 2C* and *3*). During the FU, frequent PAC and short episodes of AF could be observed in the injectable loop recorder (ILR). Therefore, she was started on antiarrhythmic drug (AAD) treatment with flecainide. During the FU with the ILR of more than 2 years after the second ablation—with additional AAD treatment—rhythm control with improved symptoms could be achieved.

Genetic testing conducted in 2021 among 11 genes (see Supplementary material online, *Data S1*) revealed a heterozygous missense variant in the *LMNA* gene: chr1:g.156136096A>G (hg38), NM_170707.4, ENST00000368300.9, c.1132A>G, and p.(Lys378Glu). This variant is absent from gnomAD v4 and from databases of published variants (HGMD, LOVD, and ClinVar). This amino acid is highly interspecies conserved up to *Tetraodon nigroviridis* (see Supplementary material online, *Figure S1*). Accordingly to the ACMG criteria, it was classified as likely pathogenic (see Supplementary material online, *Data S1*).^3^

A cardiac MRI displayed in 2022 normal ventricular cardiac dimensions and a preserved global and segmental ventricular function [LVEF 70%, right ventricular ejection fraction (RVEF) 57%] without late gadolinium enhancement (LGE).

The risk of life-threatening ventricular tachyarrhythmias (VTAs) at 5 years was estimated at 5.2% by the online calculator.^4^

### Brother of the index patient

The brother of the index patient (*Figure 1, II-1*) is an active middle-aged man at his visit in our outpatient clinic (180 cm, 77 kg) in December 2023. Persistent AF is known since the age of 25 years (*Figure 2B*). Genetic testing confirmed him to be heterozygous carrier of the *LMNA* missense variant. The patient denies complaints as palpitations or dyspnoea. No syncope. He regularly undertakes brisk walks.

A cardiac MRI displayed ventricular cardiac dimensions with preserved global and segmental ventricular function (LVEF 61%, RVEF 56%). In contrast to the MRI findings of his sister (the index patient) and his niece (daughter of the index patient), a linear layer of inferolaterobasal and inferobasal LGE was diagnosed.

The risk of life-threatening VTA at 5 years was estimated at 8.5% by the online calculator assuming a normal AV conduction despite slowly conducted AF and at 12.9% assuming a first-degree AV-block in SR.^4^

### Daughter of the index patient

The daughter of the index patient (*Figure 1, III-5*) was born in 1993. She is a young otherwise healthy female with normal height and weight (170 cm, 54 kg). She is a mother of three healthy children (twins and another child). She is asymptomatic. As her mother was diagnosed with cardiac laminopathy, cardiac screening was performed and revealed that she had inherited the *LMNA* variant from her mother.

The ECG from 2022 and 2023 demonstrated SR with frequent PAC (*Figure 2A*). A cardiac MRI was performed in October 2022. It displayed normal ventricular dimensions and a preserved global and segmental ventricular function (LVEF 65%, RVEF 54%) without LGE.

The risk of life-threatening VTA at 5 years was estimated at 6% by the online calculator.^4^

### Father of the index patient

The father of the index patient (*Figure 1, I-1*) died in 2021 in the age of 72 years during his sleep. Height and weight were normal (180 cm, 79 kg). He had a biventricular pacemaker implanted in 2014 for symptomatic bradyarrhythmia and was treated with a rivaroxaban because of long-standing persistent AF. Fourteen months prior to his death, he had developed ascites, probably caused by right cardiac congestion. The ECG from 2019 and 2020 demonstrates AF with biventricular pacing as well as premature ventricular contractions (PVC) with right bundle branch block (RBBB) and left bundle branch block (LBBB) morphology (*Figure 2D*). In March 2020, he suffered a stroke.

An echocardiography from 2020 demonstrated dilated atria, dilated LV (68 mm) with normal LVEF of 65%, and moderate mitral and tricuspid regurgitation. Pulmonary arterial systolic pressure (PAPs) was estimated at 46 mmHg.

It is unknown whether he was a carrier of the familial *LMNA* mutation. Due to the diagnosis of long-standing persistent AF in the context of the medical family history, the authors assume him as a probable *LMNA* mutation carrier.

## Discussion

This case description deals with several complex issues.

### Anticoagulation in young individuals with low thromboembolic risk score

Current guideline recommend oral anticoagulation (OAC) for all patients with clinical AF and elevated thromboembolic risk.^5^ Thromboembolic risk can be estimated using the CHADS-VASc score according to the previous guidelines,^6^ or with the modified CHADS-VA score according to the most recent 2024 guidelines.^5^ The 2024 guidelines do not provide specific recommendation for individuals with low thromboembolic risk, i.e. a CHADS-VA score = 0.^5^ However, they acknowledge that ‘the absolute risk level at which to start OAC in individual patients cannot be estimated from population-level studies’.^5^ Previous 2020 guidelines did not recommend OAC for individuals with low thromboembolic risk, i.e. a CHADS-VASc score = 0 in males and a CHADS-VASc score = 1 in females, as the benefit of preventing future thromboembolic events is perceived as low compared to the increased bleeding risk.^6^ Noteworthy, individuals with a low risk of thromboembolic risk are exposed to a low risk of bleeding as well.^6^ Furthermore, young individuals with a long exposure to a relatively low risk may have a substantial cumulative risk of thromboembolic events. There is also some evidence suggesting that an intrinsically prothrombotic atrial substrate may lead to thromboembolic events even before detection of AF.^7^ This may have been the case in the index patient. Her first stroke occurred without AF being known despite regular medical FU. It can be speculated if paroxysmal AF was not diagnosed yet before the first stroke or if atrial cardiomyopathy created a prothrombotic environment with PAC being a surrogate marker of atrial disease. Furthermore, several patient subgroups with AF are at an increased risk of ischaemic stroke and intracardiac thrombus even if treated with adequate OAC, including those with cardiac amyloidosis (CA) and hypertrophic cardiomyopathy (HCM).^5^ Therefore, current guidelines recommend OAC in patients with AF and CA or HCM, regardless of CHADS-VA score.^5^ Similarly, LMNA mutation carriers with AF may also be at risk for thromboembolic events that cannot be effectively stratified by the CHADS-VA score.

The brother of the index patient with a CHADS-VA score = 0 was not treated with OAC for more than 20 years of AF according to previous^6^ and current guideline.^5^ However, the authors of this manuscript individually believe that anticoagulation could be considered despite the low statistical risk, and the patient was recently started on direct OAC.

The daughter of the index patient is in SR with frequent PAC. There is no indication for an OAC according to current guideline recommendations.^5,7,8^ Whether OAC, outside of the current guideline recommendations, may prevent future thromboembolic events in her case can only be determined through an individualized discussion with the patient.^5,7,8^

### Rhythm or rate control in young individuals with atrial fibrillation and absent or mild symptoms

Current guidelines recommend ablation for rhythm control in patients with symptomatic AF, in patients with heart failure and AF (and as reasonable perceived benefit/risk ration), and in patients with tachyarrhythmia-induced heart failure.^6^ Asymptomatic patients and patients with only mild symptoms, as the index patient and her brother, can be treated with rate control according to the current (lack of) evidence and guidelines recommendations.^6^ Nevertheless, there is evidence of decreased risk of thromboembolic events, decreased risk of heart failure, and decreased risk of dementia in patients with SR compared to patients with AF.^1,6^ So even though younger patients may have a relatively low risk of future events on one hand, the cumulative risk for thromboembolic events, dementia, and heart failure—due to several decades of remaining life expectancy—may justify rhythm control despite the current absence of evidence and despite the lack of current guideline recommendations on this issue.^1^ On the other hand, patients with long-standing chronic AF and patients with cardiomyopathies and certain genetic conditions may have impaired outcomes in rhythm control by ablation.^6^ Two ablations and additional AAD were necessary for rhythm control in the index patient during a FU of 2 years, probably due to LMNA mutation-linked atrial cardiomyopathy. Flecainide is a class 1C AAD that has been avoided in patients with coronary artery disease and structural cardiomyopathies after the adverse outcome of the CAST trial.^9^ However, a recent study has demonstrated its safe use in patients with structural heart disease and implantable cardioverter-defibrillator (ICD).^10^ Current AF guidelines permit its use in patients with AF and no or mild heart disease.^5^

The brother of the index patient has remained without specific treatment for more than 20 years. After recent cardiological counselling, he has decided to continue rate control without specific treatment. Noteworthy, the outcome of ablation after more than 20 years of AF is probably poor. As this patient currently has a good functional status without complaints, rate control may be a reasonable strategy for him.^6^

The daughter of the index patient presented with SR with PAC in several controls. She regularly observes her heart rhythm with a smart watch. Frequent PAC and genetic arrhythmic predisposition may increase her risk of future AF with possibly adverse outcomes. Thus, prophylactic ablation of PAC may prevent or delay atrial remodelling and subsequent arrhythmic events.^8^ Currently, there is no consensus providing recommendations on medical or interventional treatment for asymptomatic PAC.^8^

### Stratification and prevention of future arrhythmic events in individuals with genetic predisposition

The risk stratification of life-threatening VTA in patients with *LMNA* mutations is important to select patients for ICD implantation.^11^ For an increasing number of cardiac conditions, a genetic diagnosis can provide prognostic information, for example, DCM due to variants in *LMNA* has an adverse prognosis and shifting recommendations towards a lower threshold for primary prevention ICD implantation, regardless of LVEF.^11^ Previous studies have reported predictors of VTA in *LMNA* genotype-positive patients including non-sustained ventricular tachycardia (nsVT), atrioventricular block (AVB), LVEF < 45%, male sex, and non-missense *LMNA* variants.^12–15^ Based on these observations, previous guidelines recommend ICD therapy in patients with *LMNA* mutations and ≥2 of these risk factors.^12,15^ A specific risk prediction model for patients with laminopathies was recently developed to facilitate the estimation of arrhythmic risk and thus indication for ICD implantation.^4,13^ A 5-year estimated risk threshold ≥ 7% predicted 96.2% of life-threatening VTA.^13^ The use of this score to guide primary prevention ICD implantation is supported by current guidelines.^11^ An external validation of the online *LMNA*-risk calculator with a registry of 118 patients from Norway and Denmark showed 83% sensitivity and 26% specificity for identifying patients with VTA during a FU of 5 years.^14^ The AVB and reduced LVEF were independent predictors of life-threatening VTA, while nsVT, male gender, and non-missense *LMNA* variants were not.^14^ In this study, the online calculator overestimated the arrhythmic risk in patients with mild and moderate phenotype, especially in male patients.^14^ Current guidelines suggest a threshold of ≥10% risk at 5 years to guide primary prevention ICD implantation in patients with DCM and *LMNA* variants^16^ and shared decision-making based on real-world data as well as individual preferences, beliefs, circumstances, and values.^11^

### Heterogeneous expression of LMNA mutations

This case report highlights atrial arrhythmias (PAC, AF) as main manifestation of a *LMNA* mutation. All patients had preserved LVEF and no evidence of VTA. Nevertheless, the father of the index patient had received a pacemaker due to bradyarrhythmia, and the brother of the index patient was diagnosed with ventricular LGE. It is therefore possible that ventricular involvement (VTA, ventricular fibrosis, systolic or diastolic dysfunction) or involvement of the conduction system occurs at a later stage of disease progression. Circulating triglyceride levels were in the normal range in all family members. This case report emphasizes therefore as well the cardio-specific character of this *LMNA* mutation as none of the *LMNA* carriers of the family had any of the other signs associated with *LMNA* mutations, i.e. particular facial features, lipodystrophy, peripheral neuronal, muscular, joint, or endocrine involvement (except hypothyroidism post-thyroiditis in the index patient).

## Conclusion

The familial occurrence of atrial arrhythmias without significant ventricular involvement in several family members with heterozygous *LMNA* missense mutation underscores the potential genetic component in cardiac arrhythmias, especially if arrhythmias occur in younger individuals without common risk factors. Individual medical and interventional treatment of the current clinical situation, including risk assessment of potential future events, genetic counselling, and regular FU surveillance with treatment adaption are essential to optimize outcomes and quality of life in families with hereditary cardiac conditions.

## Supplementary Material

ytaf129_Supplementary_Data

## Data Availability

Anonymized data that support the findings of this study are available from the corresponding author upon reasonable request.

## Lead author biography

Dr Andreas Müssigbrodt, FEHRA, FESC, is a German cardiac electrophysiologist. He graduated from the medical school in 2002 at Heidelberg University. He completed his cardiology training at Lausanne University Hospital, Marburg University Hospital, and Leipzig Heart Centre. In 2018, he submitted his habilitation thesis on the topic ‘VT Ablation in ARVC’ at the University of Leipzig and was awarded the title ‘Privatdozent’. Since 2020, he has been working as the coordinator of the electrophysiology department at the University Hospital Martinique.

## Supplementary material

**Consent:** Written informed consent was obtained from the patients to publish this report, in accordance with the COPE guidelines on ethical publishing.

**Funding:** None declared.
