# Supplementary material for: Familial cardiac laminopathy with predominant atrial involvement: a case series of a family with LMNA mutation
Source: Eur Heart J Case Rep. 2025 Mar 15;9(4):ytaf129. doi: 10.1093/ehjcr/ytaf129 (PMC11969145; doi:10.1093/ehjcr/ytaf129)
Supplement: ytaf129_Supplementary_Data [file ytaf129_supplementary_data.zip › SUPPLEMENTARY DATA.docx]

**SUPPLEMENTARY DATA**

**ACMG CRITERIA** :

PM1: located in a mutational hotspot; PM2: absent from controls; PP1 : cosegregation with disease in multiple affected family members in a gene definitively known to cause the disease; PP2: missense variant in a gene that has a low rate of missense variant and in which missense variants are a common mechanism of disease, PP3: multiple lines of computational evidence support a deleterious effect: all prediction software are concordant to consider that it is a damaging variant (SIFT, Polyphen 2 (HumDiv and HumVar), Fathmm, AlphaMissense, REVEL, ClinPred, Meta (SVM and LR) and Mistic.

**ARRHYTHMIA GENE PANEL**:

DES (NM_001927.3), DSC2 (NM_024422.3), DSG2 (NM_001943.3), DSP (NM_004415.2), FLNC (NM_001458.4), LMNA (NM_170707.2), NKX2-5 (NM_004387.3), PLN (NM_002667.3), SCN5A (NM_198056.2), TMEM43 (NM_24334.2), TNNT2 (NM_001001430.1)

**FIGURE LEGEND**

**SUPPLEMENTARY FIGURE 1** :

Protein alignment: *Homo sapiens* protein NP_733821.1 was aligned against 19 orthologues proteins: *Pan troglydytes* (H2Q091), *Mus musculus* (NP_001002011.2), *Rattus norvegicus* (G3V8L3), *Galeopterus variegatus* (XP_008568910.1), *Bos taurus* (F1MYG5), *Felis catus* (M3WFR2), Equus caballus (XP_001499938.1), *Sus scrofa* (A0A287AE06), *Loxodonta africana* (G3SR87), *Delphnaterus leucas* (A0A2Y9M085), *Balaenoptera musculus* (XP_036707496.1), *Choloepus didactylus* (XP_037681681.1), *Rhinolophys ferrumequinum* (XP_032948957.1), *Tursiops truncatus* (XP_033713208.1), *Ornithorhynchus anatinus* (F7A5P3), *Gallus gallus* (NP_990618.1), *Xenopus tropicalis* (F6YQA9), *Danio rerio* (NP_694503.1) and *Tetraodon nigroviridis* (H3CFU2).

The Lysine (K) at position 378 is indicated with a red rectangle.
