# Supplementary figures and images for: Familial cardiac laminopathy with predominant atrial involvement: a case series of a family with LMNA mutation
Source: Eur Heart J Case Rep. 2025 Mar 15;9(4):ytaf129. doi: 10.1093/ehjcr/ytaf129 (PMC11969145; doi:10.1093/ehjcr/ytaf129)

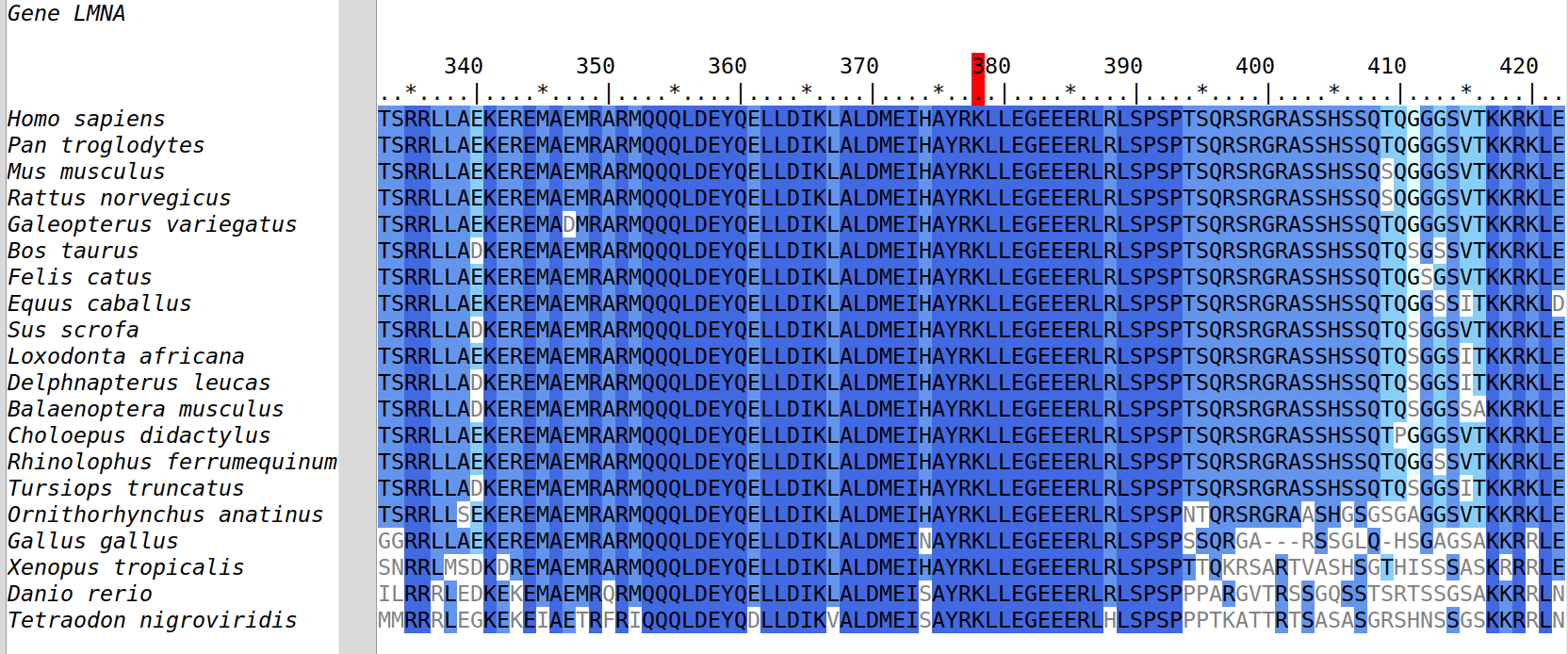

Supplement: ytaf129_Supplementary_Data [file ytaf129_supplementary_data.zip › Suppl_Fig_ProteinAlignement_240602.tif]
